# Chronic Administration of Ion Channel Blockers Impact Microglia Morphology and Function in a Murine Model of Alzheimer’s Disease

**DOI:** 10.3390/ijms241914474

**Published:** 2023-09-23

**Authors:** Ianis Kevyn Stefan Boboc, Alexandru Cojocaru, Gabriel Nedelea, Bogdan Catalin, Maria Bogdan, Daniela Calina

**Affiliations:** 1 Department of Pharmacology, University of Medicine and Pharmacy of Craiova, 200349 Craiova, Romania; 2 Experimental Research Centre for Normal and Pathological Aging, University of Medicine and Pharmacy of Craiova, 200349 Craiova, Romania; 3 U.M.F. Doctoral School Craiova, University of Medicine and Pharmacy of Craiova, 200349 Craiova, Romania; 4 Department of Physiology, University of Medicine and Pharmacy of Craiova, 200349 Craiova, Romania; 5 Department of Clinical Pharmacy, University of Medicine and Pharmacy of Craiova, 200349 Craiova, Romania

**Keywords:** Alzheimer’s disease, microglia, Verapamil, Carbamazepine, mice, behavior

## Abstract

As the population ages, a high prevalence of multimorbidity will affect the way physicians need to think about drug interactions. With microglia’s important involvement in the pathology and progression of Alzheimer’s disease (AD), understanding whether systemically administered drugs intended for other affections could impact microglia function, already impacted by the presence of beta-amyloid, is important. The aim of this study was to evaluate morphological changes of microglia, using in vivo 2-photon laser scanning microscopy, in a murine model of AD under systemic administration of sodium or calcium ion channel blockers in order to establish potential effects that these drugs might have on microglia under neuro-inflammatory conditions. A total of 30 mice (age 14–16 weeks, weight 20–25 g) were used, with 25 *APP* randomly divided into three groups. The remaining animals were *CX*_3_*CR*_1_^GFP/G^^FP^ male mice (*n* = 5) used as WT controls. After baseline behavior testing, all animals received daily intraperitoneal injections for 30 days according to the assigned group [WT (*n* = 5), Control (*n* = 5), Carbamazepine (*n* = 10), and Verapamil (*n* = 10)]. The results showed that the Verapamil treatment improved short-term memory and enhanced exploratory behavior in *APP* mice. The Carbamazepine treatment also improved short-term memory but did not elicit significant changes in anxiety-related behavior. Both Verapamil and Carbamazepine reduced the surveillance speed of microglia processes and changed microglia morphology in the cortex compared to the Control group. Due to their complex molecular machinery, microglia are potentially affected by drugs that do not target them specifically, and, as such, investigating these interactions could prove beneficial in our management of neurodegenerative pathologies.

## 1. Introduction

With longer lifespans, the number of patients treated for multiple pathologies will gradually increase [1]. This means that treatments given for one pathology could influence the progression of other afflictions coexisting in the same patient. Perhaps of all chronic pathologies, the neurodegenerative group is the least studied in terms of treatment interactions [2]. This is especially curious, as over 55% of Alzheimer’s disease (AD) patients are hypertensive [3], and some are being treated with calcium blockers. At first glance, this could be trivial. However, as calcium homeostasis alteration is one of the proposed causes of AD [4,5], the aforementioned aspect could be of significant importance. In particular, Verapamil has been reported to possess potential preventative and therapeutic properties in the context of AD [6]. Various investigations have demonstrated its broad therapeutic range, encompassing anti-inflammatory and antioxidant activities [7]. Animal studies have indicated that the administration of calcium blockers has a neuroprotective impact against neuroinflammation induced by lipopolysaccharide (*LPS*) [7]. This effect was observed through the downregulation of calcium-dependent genes associated with neuroinflammation, including interleukin-1β (*IL-1β*) and tumor necrosis factor-alpha (*TNF-α*) expression [8]. Other experimental studies have demonstrated that the modulation of voltage-gated sodium channels directly impacts the motility [8,9] and phagocytic activity [9] of microglia. This last observation shows that some drugs, although not directly targeting cells of the central nervous system (CNS), can affect their function.

With microglia heavily involved in the pathology and progression of AD [10], small alterations in their function could lead to important changes. Traditionally, this phenomenon has not been regarded as a significant issue; however, with the increased incidence of chronic pain [11,12] and high blood pressure [13,14] due to an aging population, it is not unreasonable to assume that treatments with sodium blockers and/or calcium blockers will be administered to AD patients.

Microglia morphology is directly linked to the so-called “activation” of these cells [6,15], and “activation” of microglia is one of the most important cellular findings in AD [16]. As such, in the present study, we aim to evaluate the live morphological changes of microglia using in vivo 2P-LSM in a murine model of AD and their impact on cognitive and motor function by using behavior tools [17] under systemic administration of sodium or calcium ion channel blockers.

## 2. Results

### 2.1. Verapamil Induces Increased Short-Term Memory and Exploration Behavior in APP Mice

**Figure 1:**
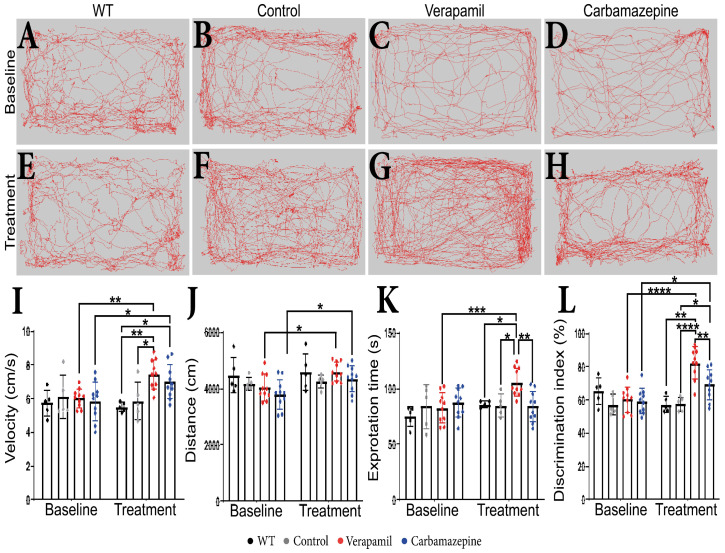
Behavioral test results. (**A**–**H**) The route traveled by the animals in OF test. (**I**) Velocity. Statistically significant difference between the Control (5.883 ± 1.123 cm/s) and Verapamil-treated (7.445 ± 0.877 cm/s) animals at 4 weeks *p* = 0.0168. Moreover, a statistically significant difference was also observed when comparing the baseline and the 4-week aftermath between the animals in the Verapamil group (*p* = 0.0053) and the animals in the Carbamazepine group (*p* = 0.0299). (**J**) Additionally, differences were highlighted when comparing the baseline and the 4-week aftermath between the groups of animals treated with Verapamil (*p* = 0.0340) and Carbamazepine, respectively (*p* = 0.0487). (**K**) Exploration time. Significant statistical differences were found between Verapamil (105.762 ± 12.638s) and Control (85.006 ± 9.902 s) animals, *p* = 0.0239, and vs. animals treated with Carbamazepine (84.005 ± 13.864 s) *p* = 0.0022, as well. Only one statistically significant difference was noted between the baseline and the 4-week aftermath in the Verapamil-treated group, *p* = 0.0008. (**L**) Novel object recognition. At 4 weeks after treatment, the group of animals treated with Verapamil spent a statistically significant amount of time near the new object compared to the group of animals treated with Carbamazepine (82.388 ± 9.460% vs. 69.281 ± 8.953%), *p* = 0.0077. Additionally, the difference between the group of animals treated with Verapamil and the Control group was statistically significant (82.388 ± 9.460% vs. 57.644 ± 4.228), *p* < 0.0001. Regarding the group of animals treated with Carbamazepine vs. the Control group, a statistically significant difference was also observed (69.281 ± 8.953 vs. 57.644 ± 4.228), with the animals of the Carbamazepine group spending more time investigating the new object, *p* = 0.00407. Between the baseline and the 4-week aftermath, a statistical difference was highlighted regarding the animals treated with Verapamil, *p* < 0.0001 and Carbamazepine, *p* = 0.0249. WT (*n* = 5); Control (*n* = 5); Carbamazepine (*n* = 10); Verapamil (*n* = 10). Results are presented as mean ± standard deviation (SD), * *p* < 0.05, ** *p* < 0.01, *** *p* < 0.001, and **** *p* < 0.0001.

No behavioral changes were detected in animals at the beginning of the experiments prior to the group assignment. During the extent of this experiment, we were not able to detect any difference between WT and Control amyloid precursor protein (*APP*) mice. Following a treatment period of 4 weeks, it was observed that *APP* mice treated with Verapamil displayed a modest increase in exploratory velocity (7.445 ± 0.877 cm/s) compared to the Control group (5.883 ± 1.123 cm/s, *p* = 0.0168) (Figure 1I). In comparison with the baseline, the *APP* mice that received the Carbamazepine treatment exhibited an increase in velocity. However, this increase was not significantly different from the rise observed in the *APP* mice treated with saline (*p* > 0.05). The administration of medication did not result in any discernible variation in the distance covered during the OF test (Figure 1H). However, animals treated with both Verapamil and Carbamazepine displayed greater distance traveled in comparison to their initial baseline measurements (Figure 1A–H,J). It is noteworthy that the administration of Verapamil resulted in a reduction in anxiety levels in mice with *APP*, as evidenced by an increase in the amount of time spent exploring the central area of the arena compared to the baseline (105.762 ± 12.638 s, *p* = 0.0008. This finding was in contrast to both the Control group (85.006 ± 9.902 s, *p* = 0.0239) and the group treated with Carbamazepine (84.005 ± 13.864 s, *p* = 0.0022) (Figure 1K). The continuous administration of Verapamil appears to enhance short-term memory in mice with *APP* pathology. Following a 4-week treatment period, mice exhibited an increased preference for the novel item (82.388 ± 9.460%) compared to the saline-treated group (57.644 ± 4.228%, *p* < 0.0001) and the Carbamazepine-treated group (69.281 ± 8.953%, *p* = 0.0077) (Figure 1L). The administration of Carbamazepine did not elicit any significant changes in anxiety-related behavior. However, it did demonstrate a positive effect on short-term memory, as evidenced by a higher percentage of memory improvement compared to the Control group (69.281 ± 8.953% vs. 57.644 ± 4.228%, *p* = 0.0407) (Figure 1L).

### 2.2. Microglia Motility and Morphology Are Sensitive to Systemic Ion Channel Blockers

**Figure 2:**
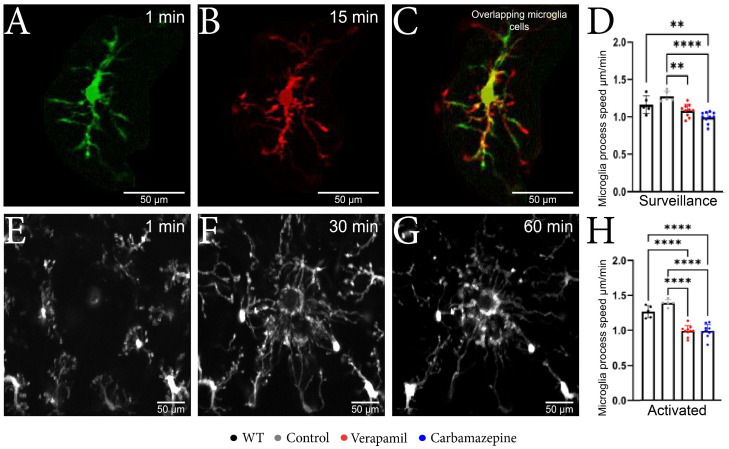
Microglial changes from the surveillance state to the active state. (**A**–**C**) Microscopic image obtained with two-photon excitation microscopy during a period of 15 min showing microglia dynamics. (**A**) Image acquired at the beginning of the experiment showing a well-defined nucleus, well-defined main branches, and few secondary branches. (**B**) Image acquired after 15 min from the beginning of the experiment where it can be observed that the nucleus is well-defined, the main branches remained with a slight elongation, and the appearance of several secondary branches. (**C**) The two overlapping microglia cells; the orange color represents the common elements; the nucleus and the fact that the main branches have remained intact can be observed; moreover, the elongation of the branches can be observed in red. (**D**) The microglia speed in the surveillance state was slower in the Verapamil-treated animals (1.085 ± 0.082 µm) vs. the Control group (1.275 ± 0.070 µm) *p* = 0.0024, but also in the Carbamazepine treated animals (0.993 ± 0.074 µm) vs. Control animals (1.275 ± 0.070 µm), *p* < 0.0001. (**E**–**G**) Microscopic image obtained after laser microlesion with two-photon excitation microscopy during a period of 60 min, highlighting the migration of the microglia towards the lesion at different time intervals: (**E**) after 1 min, (**F**) after 30 min, and (**G**) after 60 min. (**H**) In the activated state, the microglial cells present a slower movement speed in the groups treated with Verapamil (1.205 ± 0.094 µm) and Carbamazepine (1.205 ± 0.114 µm) vs. the Control group (1.692 ± 0.063 µm), *p* < 0.0001. Results are presented as mean ± SD, ** *p* < 0.01, *** *p* < 0.001, and **** *p* < 0.0001.

**Figure 3:**
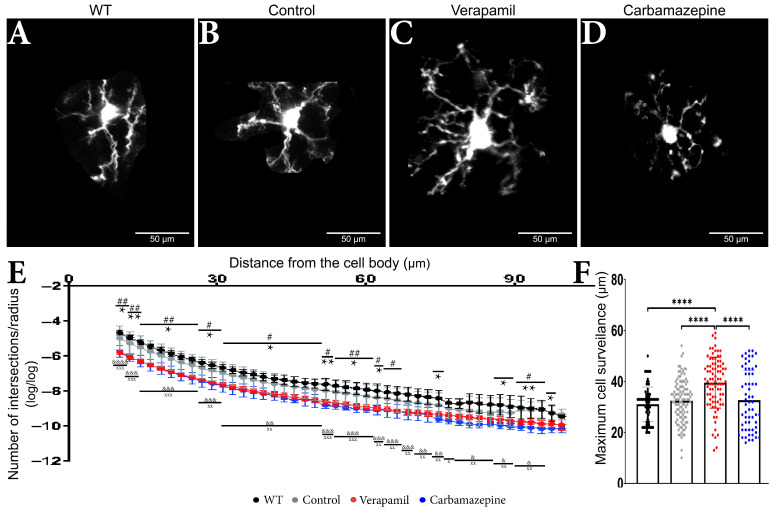
Treatment impact on the microglia’s morphology. Microglia from animals (**A**) WT, (**B**) Control, (**C**) Verapamil, and (**D**) Carbamazepine. (**E**) Number of intersections/radius. Variations in the dendritic arbor structure of microglia cells: In the 10–62 µm interval, it was observed that animals treated with Carbamazepine and Verapamil have a complex arbor structure, *p* < 0.05. In the 62–97 µm interval, statistically significant differences between the Control and treated animals were present as follows: at 66 µm (Control vs. Verapamil, *p* < 0.05), at 74 µm (Control vs. Carbamazepine, *p* < 0.05), at 87 and 89 µm (Control vs. Carbamazepine, *p* < 0.05) at 91, 93, 95 (Control vs. Verapamil, *p* < 0.05; Control vs. Carbamazepine, *p* < 0.005) and at 97 µm (Control vs. Carbamazepine, *p* < 0.05). At 99.57 µm, no statistical differences were observed. It can also be mentioned that there is no statistical difference between the Control and WT groups, (**F**) Maximum cell reach. The animals treated with Verapamil have significantly more well-arborized microglia compared to the Control group (*p* < 0.0001) but also compared to the animals treated with Carbamazepine (*p* < 0.0001). Results are presented as mean ± SD. Statistical difference between animals treated with Carbamazepine and the Control group: * *p* < 0.05, ** *p* < 0.01 and **** *p* < 0.0001. Statistical difference between animals treated with Verapamil and the Control group: # *p* < 0.05, ## *p* < 0.01. Statistical difference between animals treated with Verapamil and WT: & *p* < 0.05, && *p* < 0.01, &&& *p* < 0.001 and &&&& *p* < 0.0001. Statistical difference between animals treated with Carbamazepine vs. WT: x *p* < 0.05, xx *p* < 0.01 and xxx *p* < 0.001.

By employing in vivo 2P-LSM, we successfully demonstrated that animals treated with Verapamil and Carbamazepine displayed a reduced speed of the surveillance microglia processes (Figure 2A–C) in comparison to the Control group within the intact cortex (Figure 2D). Although the surveillance speed was slower in the Verapamil-treated animals, the cortical microglia from these mice exhibited longer surveillance distances (1.085 ± 0.082 µm) in comparison to the Control group (1.275 ± 0.070 µm, *p* = 0.0024) (Figure 3F). After the laser microlesion (Figure 2E–G), the microglia began sending their process towards the injury site, with animals treated with both Verapamil (1.205 ± 0.094 µm, *p* < 0.0001) and Carbamazepine (1.205 ± 0.114 µm, *p* < 0.0001) showing a slower process movement speed compared to the Control group (1.692 ± 0.063 µm) (Figure 2H). No statistical difference was observed between WT and *APP* mice.

Sholl measurements revealed comparable microglia morphology in the treated animals (Figure 3A–D) (Table 1). Within a range of 65 µm from the cell body, animals treated with Carbamazepine and Verapamil had distinct Sholl log–log outcomes in comparison to the Control group (Table 1) (Figure 3E,F). Occasional variations were seen between the experimental groups (Carbamazepine and/or Verapamil) and the Control group at a distance of 65 to 90 µm from the cell body within the dendritic arbor (Table 1). No statistical difference was revealed between the Control and WT groups (Table 1). At the terminal portion of the dendritic arbor, notable disparities between the experimental and control groups became evident once more (Table 1 and Figure 3E). Animals treated with Verapamil show a complex arbor structure of microglia cells with many ramified branches with a maximum reach of up to 59 µm (39.56 ± 10 µm). Microglia cells from the animals treated with Carbamazepine present a less complex arbor structure that reaches a maximum of 52 µm (32.72 ± 11.63 µm), comparable to the animals treated with saline solution 54 µm (32.46 ± 8.253 µm) (Figure 3F).

After a 20 min normal cortex imaging session, the induced laser lesion evoked an increase in microglia process speed across all groups. However, the processing speed of treated animals was lower compared to the saline-treated animals: Verapamil (1.205 ± 0.094 µm), Carbamazepine (1.205 ± 0.114 µm), Control (1.692 ± 0.063 µm), and WT (1.541 ± 0.106 µm), *p* < 0.0001.

**Figure 4:**
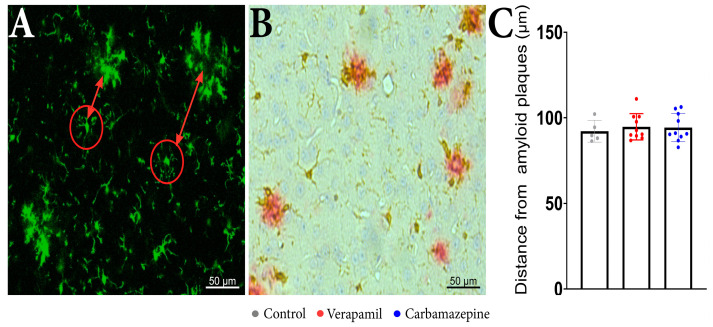
In vivo imaging of *APP* mice displays frequent microglia aggregates. (**A**) In the red circles, there are examples of the analyzed microglia cells and the distance from the microglia aggregates that are seen around beta-amyloid plaques. (**B**) IHC staining of the cortex showed Iba1^+^ (brown) and beta-amyloid plaques (red). (**C**) The distance of the analyzed cells from the beta-amyloid plaques. There were no statistically significant differences between the groups: Verapamil (94.78 ± 7.617 µm), Carbamazepine (94.29 ± 8.196 µm), and Control (92.19 ± 6.326 µm), *p* > 0.5. Control (*n* = 5); Carbamazepine (*n* = 10); Verapamil (*n* = 10). Results are presented as mean ± SD.

Regarding the distance of the analyzed cells from the beta-amyloid plaques, no statistical differences were revealed between the analyzed groups when examining the distance between the microglia aggregates (Figure 4A) and isolated cells (Figure 4C). IHC confirmed that microglia are present around amyloid plaques in the cortex of analyzed animals (Figure 4B). 

## 3. Discussion

CNS microglia, in contrast to other macrophages, display a ramified shape, where each cell occupies a distinct territory that is continuously surveyed multiple times throughout the day [18]. This process is vital for the physiological function of microglia. Because of their immune nature, microglia need to oversee an impressive number of processes, thus needing a large number of membrane receptors. If a pathological condition occurs, the microglia function needs to adapt to compensate for the imbalance, and consequently, the expression and number of receptors also change [15]. As such, microglia receptors have been linked to almost all microglia-mediated processes, such as neurodegeneration, neuroinflammation, synapse loss, and disrupted postsynaptic trafficking [19], all phenomena commonly observed in AD. With the increased aging of the world population and earlier disease detection, patients will receive longer treatments for their afflictions. Therefore, it is reasonable to assume that microglia could be susceptible to systemic treatments unrelated to CNS pathologies. With microglia functionality directly linked to their morphology [20,21], solely investigating this aspect of microglia could sometimes be sufficient to detect changes from a homeostatic steady state, as reported in aging animals [22].

We have a limited understanding of the precise ion channels that form the molecular machinery of microglia. For example, within the cerebral cortex, a multitude of splice variants of Cav1.2 have been identified [23]. However, the precise identification of any specific splice variants that may be exclusive to microglia remains uncertain. It has been suggested that microglia possess a distinct splice variation in voltage-insensitive L-type voltage-dependent calcium channels (L-VDCCs), similar to T cells [24]. This finding could perhaps account for the difficulty in detecting conventional L-VDCC currents in microglia [25], but it could also explain the contradictory results of calcium blocker therapies in AD patients, from highly advantageous to moderately detrimental [26,27,28,29]. There is also the possibility that the observed effects could not be adequately accounted for solely by neuronal effects. Therefore, in the present study, we decided to investigate if microglia morphology and surveillance are altered in a mouse model of AD receiving chronic treatments with Verapamil or Carbamazepine. We have shown that treating *APP* mice with Verapamil and Carbamazepine for 4 weeks is sufficient to elicit behavioral differences in exploration and increase short-term memory (Figure 1) in animals that have significant loads of amyloid plaques [30].

Although both Verapamil [31] and Carbamazepine [32] treatments have been reported to reduce anxiety, our findings show that only Verapamil was able to evoke enhanced exploratory behavior in *APP* animals (Figure 1K). With Verapamil blocking more than just L-Type Ca^2+^ channels [33], its use in AD should be carefully evaluated. For example, blocking the Cav3.1 T-type channel reduces non-amyloidogenic processing and generates a higher amount of Aβ peptide in an AD animal model [34]. Though the multiple pathways blocked by Verapamil and beneficial reports on animal studies, such as the present one, could mean that one of the Ca^2+^ pathways is disease-changing in AD, the individual variability in channel density and type could make a Ca^2+^-targeted therapy improbable.

While Carbamazepine has been reported to exhibit positive effects on memory and the treatment of elderly patients with seizures, as well as positive effects in the treatment of AD patients [27], in the current study, we observed modest positive changes in short-term memory in *APP* mice chronically treated with Carbamazepine (Figure 1L). Here, we were able to confirm the findings of previous reports [6] showing increased memory in *APP* animals treated with Verapamil.

The potential behavioral effects of Verapamil and Carbamazepine may arise from mechanisms involving numerous cells. However, the utilization of 2P-LSM reveals that the systemic treatment with Verapamil and Carbamazepine directly influences the morphology and functionality of microglia in the altered cortex (Figure 2H). It is noteworthy that neither of the two drugs examined in this study demonstrated any significant impact on microglial surveillance speed (Figure 2D). However, Verapamil exhibited an increase in both branching and the area under surveillance, whereas Carbamazepine just influenced branching without affecting the surveilled region (Figure 3E,F). With Carbamazepine reported to reduce microglia activation in culture models of inflammation [35,36], it could be that the morphological consequence of systemic Carbamazepine treatment is due to some CNS anti-inflammatory effect and not a consequence of AD-altering treatment. 

Understanding the changes in microglial functionality and morphology can provide insights into how systemically administered drugs could influence AD progression by altering neuroinflammation and/or neurodegenerative processes commonly seen in AD. However, investigating such results in humans should be carefully considered. If the aim of such a study is to address drug interactions, the use of Verapamil in patients under AD treatment (e.g., Aripiprazole, Galantamine, and Donepezil) should be considered because major interactions occur due to Verapamil’s inhibition of the cytochrome P450, CYP3A4 isoenzyme, thus decreasing the metabolism of these drugs [37,38]. When administered concomitantly with Rivastigmine (another drug commonly used in AD patients), bradycardia may occur (a minor interaction) through an additive effect [39]. Patients who do not have cardiovascular pathology can benefit from Verapamil therapy because the small dose administered in the preclinical stages does not affect blood pressure. In addition, patients who also present cardiac pathology and AD can benefit from this therapy as long as the occurrence of adverse reactions is monitored. Most of the concerns regarding Verapamil apply to Carbamazepine, as it can cause numerous major interactions with drugs administered in AD (Galantamine, Donepezil) by inducing the cytochrome P450 isoenzyme CYP3A4 and increasing drug metabolism [38]. Concomitant administration with Aripiprazole can produce a moderate adverse reaction, also through enzyme induction [40].

While the interaction between the two tested drugs and AD treatments is relatively well known, the use of these drugs to evaluate their potential as early altering therapies should be investigated. We would recommend their use in early onset familial AD and in the preclinical stage, using low doses. For example,80 mg/day of Verapamil, representing 1/3 of the minimum dose recommended for the treatment of cardiovascular pathologies, which is at least 240 mg/day, does not influence blood pressure [41]. Research is needed to accurately establish the full interactions and safety of systemically administered drugs in AD patients without negatively impacting its progression.

## 4. Materials and Methods

### 4.1. Animals

For this study, we crossbred *C57BL/6J-TGN* (*THY1-APKM670/671NL*; *THY1-PS1L166P*) [30] referred through this manuscript as *APP* mice to *CX*_3_*CR*_1_^GFP/GFP^ animals [42]. In total, 25 transgenic male mice (age 14-16 weeks, weight 20-25 g), positive for *APP* mutations and *CX*_3_*CR*_1_^GFP/WT^, were used. These animals were randomly divided into 3 groups (Control, *n* = 5; Carbamazepine (5 mg/kg), *n* = 10; Verapamil (3.5mg/kg), *n* = 10). Furthermore, 5 *CX*_3_*CR*_1_^GFP/WT^ male mice that were not positive for *APP* mutations, hereafter referred to as WT (age 14-16 weeks, weight 20-25 g), were used as wild-type controls. The *CX*_3_*CR*_1_^GFP^^/WT^ mutation allowed for direct in vivo visualization of microglia using 2P-LSM. During the study, all animals received food and water ad libitum and were housed in rooms with a 12-h day/night cycle from 07:00 to 19:00, with an ambient temperature of 21 °C and 60% air humidity.

### 4.2. Behavioral Studies

Behavioral testing was conducted in a manner where the examiner remained unaware of the group to which the animals were assigned. The baseline behavior recordings were made when the animals were 2 months of age, before the start of any other procedure. In order to reduce interference between tests, the Open Filed (OF) test was the first to be performed, followed by the Novel Object Recognition (NOR) test.

The OF test was performed as already described [6]. In short, locomotor and anxiety-like behavior were observed by placing the mice in the middle of a rectangular arena (15 cm height, 55 cm length, and 30 cm width) and allowing them to freely explore the environment for 10 min. The test measures various parameters such as total distance traveled, velocity, and time spent in the center of the arena.

The NOR test is widely used to assess memory and learning in mice [43]. For this study, it was performed as already described [6]. In short, the animals were placed into an arena (15 cm height, 55 cm length, and 30 cm width) with two identical objects at an equal distance (15 cm from the sidewalls) for 6 min. After this interval, animals were placed back in their home cage for 1 h. After this 1 h interval, the animals were again placed in the same arena, in which one of the identical objects was replaced with a new one. The animals were allowed to explore the new setup for 6 min. The percentage of total investigation time [time with novel object/(time with novel object + time with familiar object) × 100] was calculated and used as the basis for the existence of short-term memory [6].

All behavioral tests were recorded and analyzed using an automatic system (EthoVision XT 17, Noldus Technology, Wageningen, Netherlands). The animals were brought to the testing room 30 min before the start of the experiment. At the end of each trial, the arena was cleaned with 75% ethanol in order to remove any odor. At the end of the pharmacological treatment (see below), all animals were retested.

### 4.3. Pharmacological Treatment

Starting one week after baseline behavior testing, all animals received daily intraperitoneal injections according to the assigned group for 30 days. Control animals were given saline injections, animals in the Verapamil (Thermo Fisher Scientific, Waltham, MA, USA) group were given a dose of 3.5 mg per kilogram of body weight, whereas Carbamazepine (Thermo Fisher Scientific, Waltham, MA, USA) animals received a dose of 5 mg per kilogram of body weight.

### 4.4. Images Acquisition and Processing

After the behavior testing was concluded, the mice were anesthetized using a mix of ketamine (120 mg/kg of body mass) (Richter Pharma AG, Wels, Austria) and xylazine (12 mg/kg of body mass) (Bioveta a. s., Komenskeho, Czech Republic). After testing the depth of anesthesia, a cranial window was implanted over the right somatosensory area, as already described [44]. In order to visualize the cerebral vasculature, 10 min before placing the animal under the microscope, a subcutaneous injection of 250 nM Sulfurodamine 101 (Thermo Fisher Scientific, Waltham, MA, USA) was made [45]. The image acquisition was made using a Zeiss LSM 7 MP Multi-Photon microscope at a minimum depth of at least 150 µm consisting of a set of 50–100 images 1 µm apart, captured continuously for 20 min. After these 20-min intervals, using the laser, a microlesion was induced in the middle of the region of interest, and microglia behavior was observed for an additional 50 to 60 min. Once the acquisition was completed, images were processed using the Zen2Blue and ImageJ programs.

**Figure 5:**
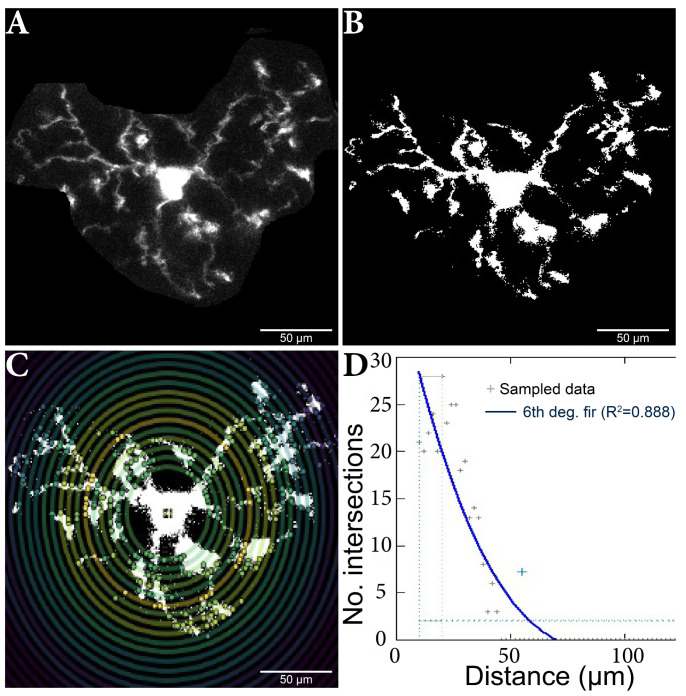
Microglia cell adjusting process and Sholl analysis output. (**A**) Original cell isolated from stack. (**B**) Binar cell after threshold process. (**C**) Cell analysis using the Sholl method; the nucleus and branch intersections can be observed, made in pairs of consecutive circles. (**D**) Sholl analysis diagram, which highlights the number of intersections in relation to the distance from the nucleus. The results were added to a database, and then the data analysis continued.

Before the cell selection and isolation, the stack was aligned using the ”Registration” [46,47] plugin, linear stack alignment with SIFT, in order to obtain good-quality images. In total, 420 cells were analyzed using the Sholl plugin for ImageJ (Figure 5) [48,49]. 

The plug was fed the maximum projection for each (manually determined) cell subjected to the (manually set) threshold adjustments. Individual cells were analyzed using the ‘’log-log” method, as shown, to detect differences in microglia morphology between the other variants [48]. The analysis started at 10 µm, a step size of 2 µm, and a maximum end radius of 300 µm.

### 4.5. Distance Measurements

In order to evaluate the distances to amyloid deposits of the analyzed microglia, we manually measured the distance from clusters of microglia that can be seen in the 2P-LSM of the *APP* mice [50,51,52]. In this regard, ImageJ software, version 1.54f., specifically the ‘Analyze Particles’ function and the ‘Multi-point’ tool for distance measurements, was used. To confirm the existence of the deposits, we also used immunohistochemistry (IHC). In short, sequential sections were cut, followed by deparaffinization and rehydration. Antigen retrieval was initially performed by microwaving the sections in citrate buffer (0.1 M, pH 6) at 650 W for 20 min. Subsequently, the sections were allowed to cool to room temperature, and endogenous peroxidase activity was inhibited using a 1% hydrogen peroxide solution for 30 min. Next, to block nonspecific binding sites, the sections were treated with a 3% skimmed milk solution (Biorad, California, CA, USA) for an additional 30 min. The slides were then incubated simultaneously with different pairs of primary antibodies as follows: rabbit anti-iba1 (1:1000, Thermo Fisher Scientific, Waltham, MA, USA) and mouse anti-Abeta (4G8 clone, 1:20,000, Signet, Dedham, MA, USA) for 18 h at 4 °C. On the following day, the signal was amplified sequentially, first with a goat anti-rabbit alkaline-phosphatase conjugated polymer (Vector Laboratories, Burlingame, CA, USA) for 1 h and then with a goat anti-mouse peroxidase-conjugated polymer for another 1 h (Vector Laboratories, Burlingame, CA, USA). After thorough washing, the signals were detected in succession, initially with Fast Red substrate for alkaline phosphatase (Vector Laboratories), followed by 3,3′-diaminobenzidine (DAB) (Vector Laboratories). Hematoxylin was used for counterstaining, and the slides were coverslipped with a glycerol-based mounting medium (Dako).

### 4.6. Statistical Analysis

Unless otherwise specified, the data were processed using the average obtained for each animal employing Prism 9 (GraphPad Software, San Diego, California, Redmond, Washington, DC, USA) and Excel (Microsoft, Redmond, WA, USA). For statistical analysis, ANOVA (Tukey’s Multiple Comparison Test) was used to ensure more power in multiple compilations. The included figures are for the individual animal value, mean value and standard deviation. If not stated otherwise, the statistical significance is indicated as follows: * *p* < 0.05, ** *p* < 0.01, *** *p* < 0.001, and **** *p* < 0.0001.

## 5. Conclusions

AD is a neurodegenerative disorder characterized by the accumulation of amyloid-beta plaques and neuroinflammation. While direct treatment of AD is still unreachable, patients suffering from this disease also require treatments for other comorbidities. With an impressive number of receptors, microglia are susceptible to long-term systemic administration of medicine. With a large interpersonal variability in the number and density of these receptors, the systemic treatment should be carefully evaluated on an individual level, and even classical drugs such as Verapamil and Carbamazepine should be under scrutiny when administered to an AD individual. 

The Verapamil treatment increased memory and exploration behavior, reduced anxiety levels, and enhanced short-term memory. Verapamil and Carbamazepine, another ion channel blocker, reduced the speed of surveillance microglia processes and affected microglia morphology in the cortex. The findings suggest that microglia morphology and surveillance are altered by ion channel blockers in the context of AD. The study highlights the potential role of ion channel blockers in modulating microglial activity and suggests their therapeutic potential in neurodegenerative conditions.

### Limitations

Limitations of the study include the use of a transgenic mouse model, which may not fully represent the complexity of human neurodegenerative diseases. The findings may not directly translate to human patients with AD. Additionally, the treatment period in the study was limited to 4 weeks. The longer-term effects of Verapamil and Carbamazepine on microglia morphology and activity were not investigated. It is possible that the observed effects may change over time.

While the study demonstrated changes in microglia morphology and activity, the underlying mechanisms behind these changes were not fully elucidated. Further research is required to understand the specific molecular pathways involved.

The study acknowledged the large interpersonal variability in the number and density of microglial receptors. This variability may impact the response to systemic treatment with ion channel blockers, making an individualized evaluation necessary. The limited sample size may restrict the generalization of the findings. The behavioral tests used in the study may have limitations in fully capturing the cognitive abilities of the animals.

## Figures and Tables

**Table 1:** Comparison of log–log Sholl analysis of microglial arbor in animals treated with Verapamil and Carbamazepine.

No.	Distance (µm)	Verapamil		Carbamazepine		Control		WT		Statistical Difference
		Mean	SD	Mean	SD	Mean	SD	Mean	SD	
1	10.3725	−5.7831	0.139557	−5.7272	0.322582	−4.97413	0.671435	−4.663	0.143934	## &&&& * xxx
2	12.447	−6.05621	0.133852	−6.07918	0.216208	−5.26317	0.662206	−4.954	0.14545	## &&& ** xxx
3	14.5215	−6.2927	0.122836	−6.2817	0.318961	−5.5213	0.653246	−5.214	0.14804	## &&& * xxx
4	16.596	−6.51083	0.11892	−6.51614	0.320295	−5.75362	0.645137	−5.448	0.15125	
5	18.6705	−6.70983	0.116308	−6.72914	0.322799	−5.96736	0.636441	−5.665	0.15497	
6	20.745	−6.89286	0.114638	−6.9243	0.326087	−6.16294	0.628662	−5.863	0.15898	
7	22.8195	−7.06227	0.113641	−7.1044	0.329918	−6.34392	0.6213	−6.046	0.16307	
8	24.894	−7.21997	0.113138	−7.27158	0.334097	−6.51256	0.614668	−6.217	0.16727	
9	26.9685	−7.36821	0.112439	−7.43002	0.340929	−6.67269	0.609749	−6.380	0.17706	# &&& * xx
10	29.043	−7.50673	0.112679	−7.57732	0.346555	−6.82148	0.6052	−6.529	0.18101	
11	31.1175	−7.63621	0.114154	−7.7148	0.351021	−6.96232	0.599641	−6.672	0.17927	# && * xx
12	33.192	−7.75639	0.1175	−7.8475	0.35843	−7.09622	0.595547	−6.807	0.18833	
13	35.2665	−7.8771	0.113572	−7.98082	0.373796	−7.22297	0.591105	−6.934	0.19231	
14	37.341	−7.99016	0.114175	−8.08488	0.365638	−7.34511	0.586263	−7.059	0.19820	
15	39.4155	−8.1003	0.114734	−8.16646	0.339129	−7.47236	0.577831	−7.196	0.21528	
16	41.49	−8.18716	0.122873	−8.27062	0.263343	−7.58284	0.573399	−7.306	0.21771	
17	43.5645	−8.26976	0.142779	−8.41186	0.206976	−7.66377	0.596221	−7.367	0.28199	
18	45.639	−8.3628	0.144326	−8.50794	0.187582	−7.76163	0.594916	−7.465	0.28714	
19	47.7135	−8.45017	0.14861	−8.49078	0.371688	−7.84599	0.588008	−7.541	0.24247	
20	49.788	−8.53619	0.150288	−8.58276	0.360576	−7.91597	0.606144	−7.586	0.24205	
21	51.8625	−8.61907	0.151902	−8.81828	0.186521	−7.98178	0.668145	−7.619	0.35071	# &&& ** xxx
22	53.937	−8.69929	0.15317	−8.86	0.170743	−8.05414	0.637683	−7.701	0.35160	## &&& * xxx
23	56.0115	−8.77759	0.154672	−8.9484	0.15765	−8.13477	0.642158	−7.777	0.35751	
24	58.086	−8.85951	0.147872	−9.0011	0.232461	−8.2165	0.642421	−7.851	0.36161	
25	60.1605	−8.93364	0.149098	−9.06893	0.185161	−8.3032	0.64599	−7.925	0.36179	
26	62.235	−8.99816	0.13772	−9.09815	0.265973	−8.36863	0.660695	−7.993	0.38311	# &&& * xx
27	64.3095	−9.0684	0.144437	−9.10358	0.401635	−8.4352	0.647236	−8.073	0.39104	# &&& xx
28	66.384	−9.1613	0.13027	−9.1714	0.404112	−8.53611	0.689189	−8.142	0.39530	
29	68.4585	−9.20471	0.151935	−9.23785	0.406829	−8.64203	0.770144	−8.203	0.39999	&&& xx
30	70.533	−9.24374	0.166534	−9.30785	0.411824	−8.68916	0.685098	−8.288	0.42385	&& xx
31	72.6075	−9.29834	0.165424	−9.361	0.415674	−8.75859	0.684737	−8.356	0.43166	
32	74.682	−9.34884	0.165499	−9.62873	0.148646	−8.8059	0.685321	−8.417	0.43390	&& * xx
33	76.7565	−9.396	0.143285	−9.7088	0.159811	−9.00829	0.580387	−8.690	0.25807	x
34	78.831	−9.45221	0.144371	−9.7904	0.167395	−9.06555	0.589108	−8.726	0.29280	& xx
35	80.9055	−9.51339	0.153587	−9.89917	0.240864	−9.13456	0.813707	−8.661	0.50328	
36	82.98	−9.56846	0.172276	−9.93613	0.204159	−9.19769	0.824955	−8.729	0.54609	
37	85.0545	−9.5962	0.181902	−9.91117	0.185086	−9.24154	0.829573	−8.783	0.55095	
38	87.129	−9.64963	0.18594	−9.9695	0.181791	−9.17946	0.770714	−8.803	0.57303	& * xx
39	89.2035	−9.7008	0.167524	−10.0297	0.176193	−9.24964	0.775958	−8.891	0.62076	
40	91.278	−9.75289	0.171649	−10.0795	0.176856	−9.06428	0.524778	−8.953	0.63885	# & ** xx
41	93.3525	−9.78566	0.159271	−10.1013	0.277616	−9.10405	0.458513	−8.994	0.54934	
42	95.427	−9.87427	0.18834	−10.1585	0.281657	−9.09462	0.450958	−9.052	0.54198	
43	97.5015	−9.88828	0.192042	−10.1612	0.222306	−9.2539	0.378871	−9.265	0.47044	*
44	99.576	−9.95285	0.307011	−10.1703	0.229126	−9.34927	0.285649	−9.478	0.23121	No Diff

No = current number, SD = standard deviation, No diff = no statistical difference. Statistical difference between animals treated with Carbamazepine vs. Control: * *p* < 0.05, ** *p* < 0.01. Statistical difference between animals treated with Verapamil vs. Control: # *p* < 0.05, ## *p* < 0.01. Statistical difference between animals treated with Verapamil vs. WT: & *p* < 0.05, && *p* < 0.01, &&& *p* < 0.001, and &&&& *p* < 0.0001. Statistical difference between animals treated with Carbamazepine vs. WT: x *p* < 0.05, xx *p* < 0.01, and xxx *p* < 0.001

## Data Availability

The data that support the findings of this study are available from the corresponding author, B.C., upon reasonable request.

## References

[1] Garin N., Koyanagi A., Chatterji S., Tyrovolas S., Olaya B., Leonardi M., Lara E., Koskinen S., Tobiasz-Adamczyk B., Ayuso-Mateos J.L. (2016). Global Multimorbidity Patterns: A Cross-Sectional, Population-Based, Multi-Country Study. J. Gerontol. A Biol. Sci. Med. Sci..

[2] Diesveld M.M.E., de Klerk S., Cornu P., Strobach D., Taxis K., Borgsteede S.D. (2021). Management of Drug-Disease Interactions: A Best Practice from the Netherlands. Int. J. Clin. Pharm..

[3] Wang J.H., Wu Y.J., Tee B.L., Lo R.Y. (2018). Medical Comorbidity in Alzheimer’s Disease: A Nested Case-Control Study. J. Alzheimers Dis..

[4] Demuro A., Mina E., Kayed R., Milton S.C., Parker I., Glabe C.G. (2005). Calcium Dysregulation and Membrane Disruption as a Ubiquitous Neurotoxic Mechanism of Soluble Amyloid Oligomers. J. Biol. Chem..

[5] Diaz J.C., Simakova O., Jacobson K.A., Arispe N., Pollard H.B. (2009). Small Molecule Blockers of the Alzheimer Abeta Calcium Channel Potently Protect Neurons from Abeta Cytotoxicity. Proc. Natl. Acad. Sci. USA.

[6] Cojocaru A., Zavaleanu A.D., Calina D.C., Osiac E., Boboc I.K.S., Mitran S.I. (2021). Different Age Related Neurological and Cardiac Effects of Verapamil on a Transgenic Mouse Model of Alzheimer’s Disease. Curr. Health Sci. J..

[7] Liu Y., Lo Y.C., Qian L., Crews F.T., Wilson B., Chen H.L., Wu H.M., Chen S.H., Wei K., Lu R.B. (2011). Verapamil Protects Dopaminergic Neuron Damage through a Novel Anti-Inflammatory Mechanism by Inhibition of Microglial Activation. Neuropharmacology.

[8] Mosalam E.M., Elberri A.I., Sallam A.S., Salem H.R., Metwally E.M., Abdallah M.S., Shaldam M.A., Mansour H.E.A. (2022). Chronotherapeutic Neuroprotective Effect of Verapamil against Lipopolysaccharide-Induced Neuroinflammation in Mice through Modulation of Calcium-Dependent Genes. Mol. Med..

[9] Guo S., Wang H., Yin Y. (2022). Microglia Polarization From M1 to M2 in Neurodegenerative Diseases. Front. Aging Neurosci..

[10] Jiang X., Wu Q., Zhang C., Wang M. (2022). Homoharringtonine Inhibits Alzheimer’s Disease Progression by Reducing Neuroinflammation via STAT3 Signaling in APP/PS1 Mice. Neurodegener. Dis..

[11] Mccarthy L.H., Bigal M.E., Katz M., Derby C., Lipton R.B. (2009). Chronic Pain and Obesity in Elderly People: Results from the Einstein Aging Study. J. Am. Geriatr. Soc..

[12] Nakai Y., Makizako H., Kiyama R., Tomioka K., Taniguchi Y., Kubozono T., Takenaka T., Ohishi M. (2019). Association between Chronic Pain and Physical Frailty in Community-Dwelling Older Adults. Int. J. Environ. Res. Public Health.

[13] Zhou B., Bentham J., Di Cesare M., Bixby H., Danaei G., Cowan M.J., Paciorek C.J., Singh G., Hajifathalian K., Bennett J.E. (2017). Worldwide Trends in Blood Pressure from 1975 to 2015: A Pooled Analysis of 1479 Population-Based Measurement Studies with 19·1 Million Participants. Lancet.

[14] Ezzati M., Zhou B., Bentham J., Di Cesare M., Bixby H., Danaei G., Hajifathalian K., Taddei C., Carrillo-Larco R.M., Djalalinia S. (2018). Contributions of Mean and Shape of Blood Pressure Distribution to Worldwide Trends and Variations in Raised Blood Pressure: A Pooled Analysis of 1018 Population-Based Measurement Studies with 88.6 Million Participants. Int. J. Epidemiol..

[15] Cojocaru A., Burada E., Bălșeanu A.T., Deftu A.F., Cătălin B., Popa-Wagner A., Osiac E. (2021). Roles of Microglial Ion Channel in Neurodegenerative Diseases. J. Clin. Med..

[16] Leng F., Edison P. (2020). Neuroinflammation and Microglial Activation in Alzheimer Disease: Where Do We Go from Here?. Nat. Rev. Neurol..

[17] Boboc I.K.S., Rotaru-Zavaleanu A.D., Calina D., Albu C.V., Catalin B., Turcu-Stiolica A. (2023). A Preclinical Systematic Review and Meta-Analysis of Behavior Testing in Mice Models of Ischemic Stroke. Life.

[18] Nimmerjahn A., Kirchhoff F., Helmchen F. (2005). Neuroscience: Resting Microglial Cells Are Highly Dynamic Surveillants of Brain Parenchyma in Vivo. Science (80-).

[19] Schürmann B., Bermingham D.P., Kopeikina K.J., Myczek K., Yoon S., Horan K.E., Kelly C.J., Martin-de-Saavedra M.D., Forrest M.P., Fawcett-Patel J.M. (2020). A Novel Role for the Late-Onset Alzheimer’s Disease (LOAD)-Associated Protein Bin1 in Regulating Postsynaptic Trafficking and Glutamatergic Signaling. Mol. Psychiatry.

[20] Stopper L., Bălşeanu T.A., Cătălin B., Rogoveanu O.C., Mogoantă L., Scheller A. (2018). Microglia Morphology in the Physiological and Diseased Brain—From Fixed Tissue to in Vivo Conditions. Rom. J. Morphol. Embryol..

[21] Morrison H., Young K., Qureshi M., Rowe R.K., Lifshitz J. (2017). Quantitative Microglia Analyses Reveal Diverse Morphologic Responses in the Rat Cortex after Diffuse Brain Injury. Sci. Rep..

[22] Godeanu S., Clarke D., Stopper L., Deftu A.F., Popa-Wagner A., Bălșeanu A.T., Scheller A., Catalin B. (2023). Microglial Morphology in the Somatosensory Cortex across Lifespan. A Quantitative Study. Dev. Dyn..

[23] Clark M.B., Wrzesinski T., Garcia A.B., Hall N.A.L., Kleinman J.E., Hyde T., Weinberger D.R., Harrison P.J., Haerty W., Tunbridge E.M. (2020). Long-Read Sequencing Reveals the Complex Splicing Profile of the Psychiatric Risk Gene CACNA1C in Human Brain. Mol. Psychiatry.

[24] Kotturi M.F., Jefferies W.A. (2005). Molecular Characterization of L-Type Calcium Channel Splice Variants Expressed in Human T Lymphocytes. Mol. Immunol..

[25] Hopp S.C. (2021). Targeting Microglia L-Type Voltage-Dependent Calcium Channels for the Treatment of Central Nervous System Disorders. J. Neurosci. Res..

[26] Zhang Y., Zhang L., Zhou C.-N., Jiang L., Tang J., Liang X., Fan J.-H., Dou X.-Y., Tang Y. (2020). Oligodendrocyte Generation and Maturation in the Hippocampus May Be a Target of Fluoxetine for Delaying Cognitive Dysfunction during Early Alzheimer’s Disease. arXiv.

[27] Hoyt C.T., Domingo-Fernández D., Balzer N., Güldenpfennig A., Hofmann-Apitius M. (2018). A Systematic Approach for Identifying Shared Mechanisms in Epilepsy and Its Comorbidities. Database.

[28] Wang C.C., Kong J.Y., Li X.Y., Yang J.Y., Xue C.H., Yanagita T., Wang Y.M. (2022). Antarctic Krill Oil Exhibited Synergistic Effects with Nobiletin and Theanine in Ameliorating Memory and Cognitive Deficiency in SAMP8 Mice: Applying the Perspective of the Sea–Land Combination to Retard Brain Aging. Front. Aging Neurosci..

[29] Wang C., Huang W., Lu J., Chen H., Yu Z. (2022). TRPV1-Mediated Microglial Autophagy Attenuates Alzheimer’s Disease-Associated Pathology and Cognitive Decline. Front. Pharmacol..

[30] Radde R., Bolmont T., Kaeser S.A., Coomaraswamy J., Lindau D., Stoltze L., Calhoun M.E., Jäggi F., Wolburg H., Gengler S. (2006). Aβ42-Driven Cerebral Amyloidosis in Transgenic Mice Reveals Early and Robust Pathology. EMBO Rep..

[31] (1988). Verapamil Appears to Reduce Anxiety and Panic Attacks. InPharma.

[32] Uhde T.W., Ballenger J.C., Post R.M. (1985). Carbamazepine: Treatment of Affective Illness and Anxiety Syndromes. Psychiatry State Art.

[33] Kuryshev Y.A., Brown A.M., Duzic E., Kirsch G.E. (2014). Evaluating State Dependence and Subtype Selectivity of Calcium Channel Modulators in Automated Electrophysiology Assays. Assay Drug Dev. Technol..

[34] Rice R.A., Berchtold N.C., Cotman C.W., Green K.N. (2014). Age-Related Downregulation of the CaV3.1 T-Type Calcium Channel as a Mediator of Amyloid Beta Production. Neurobiol. Aging.

[35] Ismail F.S., Corvace F., Faustmann P.M., Faustmann T.J. (2021). Pharmacological Investigations in Glia Culture Model of Inflammation. Front. Cell. Neurosci..

[36] Franklin R.J.M., Ffrench-Constant C., Edgar J.M., Smith K.J. (2012). Neuroprotection and Repair in Multiple Sclerosis. Nat. Rev. Neurol..

[37] Cruz M.P. (2016). Aripiprazole Lauroxil (Aristada): An Extended-Release, Long-Acting Injection For the Treatment of Schizophrenia. Pharm. Ther..

[38] Zhou S.-F. (2008). Drugs Behave as Substrates, Inhibitors and Inducers of Human Cytochrome P450 3A4. Curr. Drug Metab..

[39] Jang H.L., Hyeon M.R., Myung H.B., Yong S.K., Ju H.L., Yongwhi P., Heo J.H., Young S.L., Dong H.Y., Hun S.P. (2009). Prognosis and Natural History of Drug-Related Bradycardia. Korean Circ. J..

[40] FDA 2002 Highlights of Prescribing Information Abilify® (Aripiprazole). https://www.accessdata.fda.gov/drugsatfda_docs/label/2014/021436s038,021713s030,021729s022,021866s023lbl.pdf.

[41] Ahmed H., Ishrat T. (2022). Repurposing Verapamil for Prevention of Cognitive Decline in Sporadic Alzheimer’s Disease. Neural. Regen. Res..

[42] Jung S., Aliberti J., Graemmel P., Sunshine M.J., Kreutzberg G.W., Sher A., Littman D.R. (2000). Analysis of Fractalkine Receptor CX(3)CR1 Function by Targeted Deletion and Green Fluorescent Protein Reporter Gene Insertion. Mol. Cell. Biol..

[43] Lueptow L.M. (2017). Novel Object Recognition Test for the Investigation of Learning and Memory in Mice. J. Vis. Exp..

[44] Cătălin B., Mitran S., Albu C., Iancău M. (2013). Comparative Aspects of Microglia Reaction in White and Gray Matter. Curr. Health Sci. J..

[45] Nimmerjahn A., Kirchhoff F., Kerr J.N.D., Helmchen F. (2004). Sulforhodamine 101 as a Specific Marker of Astroglia in the Neocortex in Vivo. Nat. Methods.

[46] Lowe D.G. (2004). Distinctive Image Features from Scale-Invariant Keypoints. Int. J. Comput. Vis..

[47] Registration. https://imagej.net/imaging/registration.

[48] Cătălin B., Alexandru D., Albu C., Iancău M. (2013). Original Paper Microglia Branching Using a Sholl Analysis Method. Curr. Health Sci. J..

[49] Sholl Analysis. https://imagej.net/plugins/sholl-analysis.

[50] Jung C.K.E., Keppler K., Steinbach S., Blazquez-Llorca L., Herms J. (2015). Fibrillar Amyloid Plaque Formation Precedes Microglial Activation. PLoS ONE.

[51] Huang Y., Happonen K.E., Burrola P.G., O’Connor C., Hah N., Huang L., Nimmerjahn A., Lemke G. (2021). Microglia Use TAM Receptors to Detect and Engulf Amyloid β Plaques. Nat. Immunol..

[52] Hierro-Bujalance C., Bacskai B.J., Garcia-Alloza M. (2018). In Vivo Imaging of Microglia with Multiphoton Microscopy. Front. Aging Neurosci..

